# Perceived Social Support Moderates the Link between Attachment Anxiety and Health Outcomes

**DOI:** 10.1371/journal.pone.0095358

**Published:** 2014-04-15

**Authors:** Sarah C. E. Stanton, Lorne Campbell

**Affiliations:** Department of Psychology, University of Western Ontario, London, Ontario, Canada; City University of Hong Kong, Hong Kong

## Abstract

Two literatures have explored some of the effects intimate relationships can have on physical and mental health outcomes. Research investigating health through the lens of attachment theory has demonstrated that more anxiously attached individuals in particular consistently report poorer health. Separate research on perceived social support (e.g., partner or spousal support) suggests that higher support has salutary influences on various health outcomes. Little to no research, however, has explored the interaction of attachment anxiety and perceived social support on health outcomes. The present study examined the attachment-health link and the moderating role of perceived social support in a community sample of married couples. Results revealed that more anxious persons reported poorer overall physical and mental health, more bodily pain, more medical symptoms, and impaired daily functioning, even after controlling for age, relationship length, neuroticism, and marital quality. Additionally, perceived social support interacted with attachment anxiety to influence health; more anxious individuals' health was poorer even when perceived social support was high, whereas less anxious individuals' health benefited from high support. Possible mechanisms underlying these findings and the importance of considering attachment anxiety in future studies of poor health in adulthood are discussed.

## Introduction

Every year millions of individuals suffer myriad health problems ranging from being experienced as a nuisance to life-threatening. Interestingly, individual differences and interpersonal processes have been identified by researchers as important sources of health problems [1], [2]. One specific theoretical framework that takes into account individual differences in how people perceive and relate to close others is attachment theory. Attachment anxiety in particular has been linked to acute physiological responses to stress [3], [4], as well as poorer health and more illness [5]–.

Research on intimate relationships and health has also highlighted the importance of adequate perceived social support in buffering against deleterious mental and physical health outcomes [8], [9]. More anxiously attached individuals, however, have biased and often negative perceptions of social support [10], [11]. These negative perceptions of social support, combined with acute physiological bodily responses to stress, may therefore undermine the potential salutary effects of social support on health for more anxious persons specifically; that is, more anxious persons' health may suffer regardless of whether or not they feel they have adequate social support [12].

Although an increasing number of studies have examined attachment and acute physiological (e.g., cortisol) responses to stress through a dyadic lens, the majority of attachment and physical and mental health research has examined samples of undergraduate students or patients, and has not collected data from dyads. Additionally, the potential moderating role of perceived social support on the attachment-health link has received little attention from scholars to date. The present study was therefore designed to expand understanding of the attachment-health link. We first present an overview of attachment theory, with a particular focus on the current literature on attachment anxiety and health outcomes and the potential for investigations of perceived social support to help clarify the attachment-health link. We then outline the present research, which examined the relation between attachment anxiety, perceived social support, and health outcomes in a large community sample of married couples.

### Attachment Theory

According to Bowlby [13]–[15], early interactions with significant others generate internal working models of the self and others that guide behavior and influence perceptions about what relationships should be like [16]. Over the years, researchers have suggested that two dimensions tap individual differences in adult attachment [17]. The *avoidance* dimension reflects the extent that individuals feel uncomfortable with closeness and intimacy in their relationships. People scoring higher on attachment avoidance tend to be less invested in their relationships and try to remain emotionally independent of their partners [18]. The *anxiety* dimension reflects the extent that individuals worry and ruminate about rejection or abandonment from their partners. People scoring higher on attachment anxiety tend to crave affection from their partners while simultaneously distrusting their partners' love [19]. Secure individuals score low on both attachment anxiety and attachment avoidance; they are comfortable with intimacy and do not fear abandonment by their partners.

Mikulincer and Shaver [20], [21] explored the activation and operation of the adult attachment system, specifying that the primary attachment system strategy is seeking proximity to significant others during times of need. Attachment *security* develops when attachment figures are consistently available and responsive. On the other hand, if attachment figures are consistently unavailable or unresponsive attachment *insecurity* develops, resulting in the use of one of two secondary attachment strategies (given that the primary strategy of proximity seeking is ineffective). *Deactivating* strategies involve the inhibition of proximity seeking, typical of individuals scoring higher on attachment avoidance. Thus, more avoidant individuals seek to maintain independence and self-reliance, denying needs or emotional states that might activate the attachment system [21]. In contrast, *hyperactivating* strategies involve making stronger attempts to seek proximity in order to gain attention, care, and support, typical of individuals scoring higher on attachment anxiety. Thus, more anxious individuals monitor their relationship partners closely for signs indicating waning interest and closeness [22].

A result of this chronic monitoring for rejection from their partners is that more anxiously attached individuals have a low threshold for perceiving events in the environment as threatening to the relationship [21]. For example, more anxious individuals tend to perceive more conflict in their relationships and escalate conflict severity [23], assign negative interpretations to their partners' behavior [11], and manage distress by attending to its source in a hypervigilant manner [24]. More anxious individuals' chronic tendencies to worry and ruminate about their relationships, therefore, may undermine their physical and mental health outcomes. Indeed, as discussed in the following section, the hyperactivation of the attachment system for more anxious individuals seems to be not only psychological, but also physiological.

### Attachment Anxiety and Health

Physiological arousal associated with more anxious individuals' frequent attachment system activation is particularly acute [12], [25]. Specifically, more anxiously attached individuals exhibit greater hypothalamic-pituitary-adrenocortical (HPA) axis and autonomic nervous system (ANS) reactivity to stressful events [25]. For instance, more (vs. less) anxiously attached individuals demonstrate increased cortisol reactivity when experiencing general, non-relationship stress [4], as well as relationship-specific stress such as conflict [26]. Following such stress (particularly if relationship-relevant), the cortisol levels of more anxious individuals take longer to return to baseline.

Unsurprisingly, then, given researchers' assumptions that acute biological responses to stress can negatively influence general health, attachment anxiety has been linked with physical and mental health problems [12]. More anxious individuals exhibit higher blood pressure [3] and heart rate [27] after stressful or unpleasant interactions with others, and report experiencing more physical symptoms indicative of ill health [5]. Higher attachment anxiety is also associated with risky health behavior [7] and sleep problems [28]. The negative self and other perceptions more anxious persons hold may therefore “prime” them to experience and be affected by stress. That is, it seems that more anxious individuals invest heavily in monitoring their environment for rejection, at the expense of their health.

### Attachment Anxiety and Social Support

Social support has long been linked to positive health outcomes [8], [9]. For example, individuals who perceive greater social support tend to have lower mortality rates from disease [29]. However, attachment research has shown that more anxiously attached individuals tend to have biased perceptions of social support. For example, Collins and B. C. Feeney [11] found that more anxious individuals generally perceive less support from their partners and often remember a partner's helpful behavior more negatively. Nonetheless, when more anxious individuals *do* perceive support from their romantic partners, they experience greater relationship quality and other positive psychological outcomes over time [30], [31]. These latter findings suggest, then, that higher perceived social support may be *psychologically* soothing for more anxious persons.

To be sure, social support can have salutary influences on health. However, when individuals experience augmented reactions to all types of stress (as is the case with attachment anxiety), the benefits of social support may not be enough to improve health outcomes. In other words, although more anxiously attached persons may benefit *psychologically* from social support, the advantages associated with support may not carry over to their health outcomes because such outcomes are undermined *physiologically* [12]. That is, because more anxious individuals in particular exhibit acute bodily reactions to stress [4], [26], which in turn may directly impact health, they may experience poorer health regardless of social support. Recent studies provide preliminary support for this notion; following stress, more anxious persons have greater cortisol reactivity [32] and slower cortisol recovery [33] regardless of whether their perceived level of social support was low or high. Nevertheless, in one study higher perceived social support did reduce negative feelings following stress [32]. These findings suggest that perceived social support may alleviate psychological, but not physiological, responses to stress. We propose, therefore, that perceived social support should not benefit more anxious persons' physical and mental health outcomes.

### The Present Research

The extant research on attachment anxiety and health outcomes has some important limitations. First, this small body of research has primarily focused on samples of undergraduate university students (a relatively healthy population) or patients (a relatively unhealthy population). Moreover, this research has assessed individuals exclusively, as opposed to both members of the dyad. An additional shortcoming is that most prior research has measured health outcomes using relatively simple measures (e.g., checklists). Finally, although recent research suggests that adequate perceived social support can have soothing effects on psychological (e.g., negative affect), but not physical (e.g., cortisol), outcomes for more anxious persons, these studies have explored such effects only in relation to acute bodily reactions (e.g., HPA pathway hormones). Thus, little to no research has examined the possible moderating role social support may play in the health outcomes for individuals high or low in anxious attachment. Indeed, the social support-health and attachment-health links are examined largely independently. Research examining both partners, utilizing community samples and well-validated and diverse measures of long-term health outcomes, that also investigates social support would allow for greater generalization of the link between attachment anxiety and health.

The present study was designed to more extensively explore the attachment-health link. We first predicted that more (vs. less) anxiously attached individuals would report more negative health symptoms and poorer overall health on a number of dimensions (e.g., pain). Next, since highly anxious individuals worry and ruminate about their close relationships, we predicted that more anxious individuals would report that their health impaired their daily functioning (e.g., social functioning). We also expected higher social support (regardless of attachment orientations) to be associated with positive health outcomes, consistent with prior research [9]. Lastly, we expected social support to moderate the attachment-health link such that when individuals perceive more available social support, the health benefits would extend primarily to those less anxiously, and not more anxiously, attached, conceptually consistent with prior research [32], [33].

We explored the possibility that there are links between Partner A's anxious attachment and Partner B's health outcomes, but we did not make specific hypotheses a priori. There is some evidence to suggest that romantic partners may influence each other's health; for example, couples' cortisol levels have been shown to coregulate [34]. Nonetheless, little research has examined such coregulation in the context of attachment theory and we did not have strong theoretical reasons to expect particular partner effects to occur. Meuwly et al. [33], for instance, did not find consistent partner effects in their study of attachment, social support, and physiological stress. Thus, we treated partner effect analyses as exploratory. We also did not make a priori hypotheses about attachment avoidance, as some research on attachment avoidance and health finds effects such that more avoidant persons experience poorer health, but other research does not find these links [12].

## Method

### Ethics Statement

This research study was approved by the University of Western Ontario institutional review board (IRB). The rights of participants were protected and written informed consent was obtained.

### Participants

A sample of 116 heterosexual married couples was taken from a large community in southwestern Ontario, Canada using advertisements in local newspapers. The average age was 38.6 years for men (*SD* = 11.2 years) and 36.7 years for women (*SD* = 10.7 years), and marriage length ranged from 2 months to 53 years (*M* = 10.1 years, *SD* = 10.6 years). Participants received $50.00 CDN each for completing the study.

### Materials and Procedure

Couples attended a two-hour laboratory session and separately and privately completed a booklet of questionnaires. To assess attachment orientations, participants completed the Experiences in Close Relationships-Revised Questionnaire (ECR-R; [35]), a 36-item questionnaire containing 18 items measuring attachment avoidance (e.g., “I get uncomfortable when a romantic partner wants to be very close”) and 18 items measuring attachment anxiety (e.g., “I often worry that my partner doesn't really love me”). Participants rated each item on a 7-point Likert scale (1 =  *strongly disagree*, 7 =  *strongly agree*). Avoidance scores were created by averaging responses across the avoidance dimension items, α = .93 (men), α = .94 (women). Anxiety scores were created by averaging responses across the anxiety dimension items, α = .89 (men), α = .89 (women).

Participants reported on their health using two measures. The first was a medical symptoms list containing a subset of the 126 medical conditions varying in severity (e.g., “digestive upsets”) from the Serious of Illness Rating Scale (SIRS; [36]). Women responded to 38 medical symptoms (including menstrual problems), whereas men responded to 37 medical symptoms (not including menstrual problems). Participants placed a checkmark beside symptoms they were subject to at the present time, and the number of symptoms reported was summed for each participant. (Scores were standardized within gender to take into account the different number of symptoms on the checklists for men and women.)

The second health scale was Stewart, Hays, and Ware's [37] 20-item MOS Short-form General Health Survey. This measure contains six subscales assessing (1) pain (“How much bodily pain have you had during the past four weeks?”), (2) overall health perceptions (e.g., “I am as healthy as anybody I know”), (3) social functioning (“How much of the time during the past month has your health limited your social activities, like visiting with friends?”), (4) physical functioning (e.g., “How long (if at all) has your health limited you in the activities you can do, like moving a table, carrying groceries, or bowling?”), (5) role functioning (e.g., “Does your health keep you from working at a job, doing work around the house, or going to school?”), and (6) mental health (e.g., “How much of the time during the past month have you felt downhearted and blue?”). Subscales were rated on Likert scales and all demonstrated acceptable reliability statistics for men and women (αs ranged from .82 to .89).

To assess social support, participants completed a version of the Social Provisions Scale (SPS; [38]) comprising 12 items asking whether they feel they can turn to their romantic partner (in this study, their spouse) during times of need (e.g., “Can you depend on your spouse to help you when you really need it?”), rated on a 3-point scale (1 =  *no*, 2 =  *sometimes*, 3 =  *yes*), α = .78 (men), α = .83 (women).

Participants also completed the satisfaction subscale of the Dyadic Adjustment Scale (DAS; [39]) to assess the extent to which they were happy in their marriage (e.g., “Do you ever regret that you married?”), and responses were measured on a 6-point Likert scale (1 =  *all the time*, 6 =  *never*), α = .80 (men), α = .90 (women).

Finally, on a 5-point scale (1 =  *disagree strongly*, 5 =  *agree strongly*), participants reported their level of neuroticism on seven items (e.g., “I see myself as someone who can be tense,”) from the Big Five Inventory (BFI; [40]), α = .89 (men), α = .79 (women). After completing all questionnaires, participants were debriefed and dismissed.

## Results

For descriptive purposes the means and standard deviations of all primary study variables for men and women, as well as the correlations of study variables for men and women and also between partners, are presented in Table 1. The zero-order correlations show that more anxiously attached individuals (particularly women) reported more medical symptoms. More anxious individuals also reported lower perceived social support. Additionally, more anxious individuals reported poorer overall health perceptions, poorer social functioning, and more mental distress (i.e., poorer mental health). Correlations between spouses emerged such that when an individual reported more symptoms, his/her partner did as well. Furthermore, when an individual reported better perceptions of overall health, better social and role functioning, better mental health, and greater perceived social support, so did his/her partner.

**Table 1:** Means, Standard Deviations, and Correlations among Predictor and Outcome Measures.

Variable	1	2	3	4	5	6	7	8	9	10	Mean	Std Dev
1 Attachment Anxiety	**.26** **	.67**	.12	.12	−.16†	−.16†	−.04	−.07	.35**	−.62**	1.90	0.75
2 Attachment Avoidance	.64**	**.44** **	.06	.02	−.15†	−.04	−.06	−.02	.30**	−.64**	2.16	0.92
3 Number of Symptoms	.43**	.25**	**.29** **	.41**	−.53**	−.38**	−.56**	−.49**	.48**	−.12	2.61	2.99
4 Pain	.16†	.01	.48**	**.12**	−.51**	−.35**	−.26**	−.45**	.31**	−.05	2.56	1.19
5 Health Perceptions	−.34**	−.23*	−.43**	−.45**	**.32** **	.50**	.46**	.66**	−.51**	.09	4.05	0.82
6 Social Functioning	−.20*	−.15	−.37**	−.44**	.64**	**.24** **	.26**	.56**	−.48**	.07	5.72	0.69
7 Physical Functioning	.06	.03	−.28**	−.47**	.48**	.50**	**.05**	.67**	−.19*	−.07	2.84	0.33
8 Role Functioning	−.12	−.05	−.32**	−.35**	.68**	.63**	.59**	**.21** *	−.42**	−.01	2.79	0.52
9 Mental Health	.52**	.34**	.49**	.14	−.33**	−.26**	.02	−.18*	**.34** **	−.38**	2.08	0.78
10 Perceived Social Support	−.68**	−.68**	−.28**	−.03	.18*	.06	−.04	.05	−.42**	**.42** **	0.02	0.24
Mean	1.93	2.44	3.61	2.47	3.95	5.54	2.68	2.73	2.08	−0.02		
Std Dev	0.89	0.79	3.10	1.22	0.97	0.94	0.50	0.56	0.76	0.30		

*Note*. Correlations below the diagonal are for women, whereas correlations above the diagonal are for men. Correlations between spouses appear along the diagonal.

† *p*<.10,

*p<.05,

**p<.01.

The data analytic approach to test primary study hypotheses was influenced by the Actor-Partner Interdependence Model (APIM) [41]. Including both actor and partner effects in the model allows the testing of mutual influence that may occur between individuals within a relationship, and controls for variance in individuals' outcome variable scores that could be associated with their partner's predictor variable scores. We tested our models using hierarchical linear modeling (HLM) [42], following the suggestions of Campbell and Kashy [43] regarding the use of HLM for dyadic data. In the case of dyadic data, HLM treats the data from each partner as nested scores within a group that has *n* = 2.

Gender was effect coded (−1 =  men, 1 =  women), and all continuous predictor variables were centered on the grand mean. Interaction terms of gender with the actor and partner effects of attachment anxiety and avoidance were originally entered into the models, but no gender differences were found. Therefore, the results for the models are presented pooled across gender.

We ran seven models, one for each self-reported health outcome. The actor and partner effects of attachment anxiety and avoidance were included as predictors in each equation, as was gender. In a second step, we included actor and partner effects for social support, as well as the interaction between actor attachment anxiety and actor social support to test for moderation effects. Results from the mixed model analyses are presented in Table 2.

**Table 2:** Results from Mixed Models with Actor and Partner Scores on Attachment Anxiety and Avoidance, Gender, and Social Support Predicting Health Outcomes.

Predictor Variable	Number of Symptoms	Pain	Health Perceptions	Social Functioning	Physical Functioning	Role Functioning	Mental Health
Step 1							
Attachment Anxiety							
Actor	.42**	.33**	−.28**	−.23**	.02	−.08	.39**
Partner	.06	−.09	−.01	−.06	.04	−.06	.10
Attachment Avoidance							
Actor	−.09	−.17	−.06	.03	−.03	.04	.03
Partner	−.05	.04	.09	.09	−.01	.01	−.05
Gender	.01	−.02	−.02	−.08	−.08**	−.04	−.02
Step 2							
Social Support							
Actor	−.32	−.65	.33	.22	.72*	.89*	−.59*
Partner	−.13	−.41	.19	−.16	.05	.01	−.08
Actor Attachment Anxiety × Actor Social Support	.12	.33	−.58**	−.49*	−.29**	−.35**	.05

*Note*. Reported values are unstandardized regression coefficients. Significance levels are given for each predictor variable at the initial point of entry in the regression equation.

*p<.05,

**p<.01.

### Attachment Anxiety and Health Outcomes

Main effects of gender emerged in only one analysis, with husbands reporting that their health impaired their physical functioning more so than wives. Significant actor effects of anxious attachment emerged in five models. As expected, and consistent with prior research, more (vs. less) anxious individuals reported currently experiencing more medical symptoms. Additionally, more anxious individuals reported experiencing more pain, more negative overall health perceptions, and that their health impaired their social functioning. Lastly, more anxious individuals reported experiencing more mental distress (e.g., greater depression).

No actor effects of attachment avoidance emerged in any of the models tested. Additionally, no partner effects of attachment anxiety or attachment avoidance emerged. For exploratory purposes we entered the interaction term between anxious and avoidant attachment in our models, but no significant interactions emerged. In our sample, therefore, the number of medical symptoms currently being experienced and self-reported health was primarily linked to individuals' own degree of attachment anxiety.

### Attachment Anxiety and Social Support

Significant actor effects of social support emerged in three models. Individuals who perceived higher (vs. lower) social support in their marriage reported better physical and role functioning, and less mental distress. No partner effects of social support emerged.

**Figure 1:**
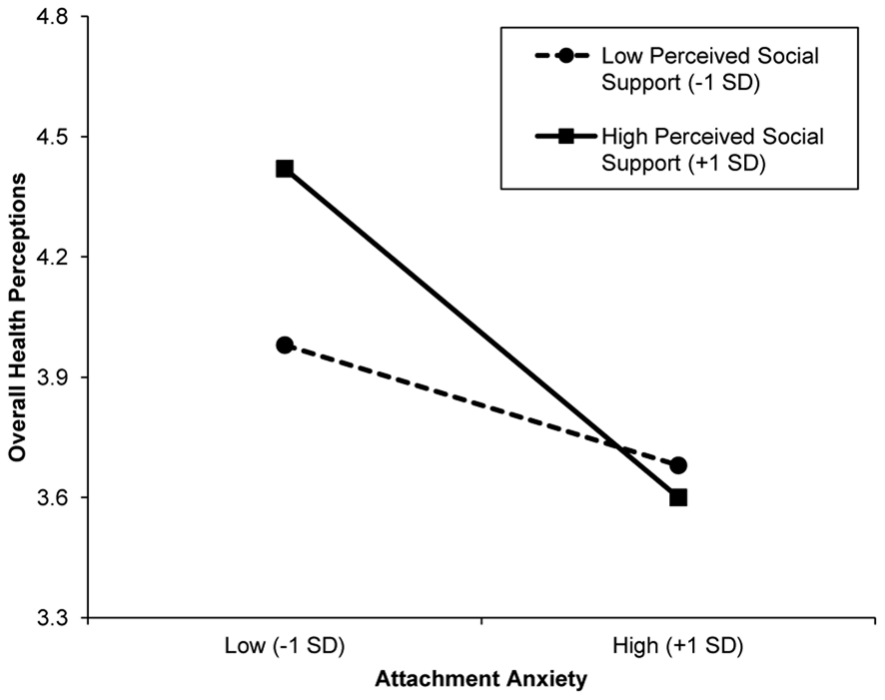
Moderation of the attachment anxiety effect on health perceptions by perceived social support. Low/high attachment anxiety and low/high perceived social support represent ±1 standard deviation of the mean.

As predicted, actor social support moderated the attachment-health link in four models. Specifically, when social support was low, more and less anxiously attached individuals reported similar overall health perceptions, social, and role functioning, *p*s>.10. When social support was high, however, more (vs. less) anxious individuals reported poorer overall health perceptions, and worse social and role functioning, *b* = −0.49, *t*(215)  = −4.12, *p*<.001, *b* = −0.42, *t*(219)  = −3.69, *p*<.001, and *b* = −0.21, *t*(219)  = −2.79, *p* = .006, respectively. The pattern of interaction for physical functioning was similar, but tests of simple effects were not statistically significant, *p*s>.10. Overall, less anxious individuals reported more positive health outcomes when social support was relatively high, whereas more anxious individuals did not appear to derive health benefits from perceiving high levels of social support. An illustrative graph for overall health perceptions is displayed in Figure 1 (the pattern of interaction was identical for each significant outcome variable).

### Discriminant Analyses

We included several control variables in follow-up analyses. Age is certainly a factor related to health, and in our sample we had some participants in their 20 s and others in their 50 s. Additionally, there is a component of neuroticism in attachment anxiety, and neuroticism has also been linked to poor health [44]. It could be that greater age or higher neuroticism could explain the health effects that emerged in our models. We also included marital quality and relationship length as possible control variables. We re-ran all of the models presented in Table 2 controlling for actor and partner age, relationship length, neuroticism, and marital quality scores, separately. The results from these discriminant analyses are available as supplementary material (Tables S1–S4). Importantly, when controlling for these variables the actor effects of anxious attachment and their interactions with social support remained robust.

## Discussion

In the present research, consistent links were found between attachment anxiety and health outcomes in a large community sample of married couples. Specifically, more (vs. less) anxiously attached individuals reported more medical symptoms, more bodily pain, and poorer overall health, in addition to reporting that their health negatively impacted their ability to function in their lives (e.g., social functioning). Furthermore, the present study introduced the novel finding that perceived social support moderated the attachment-health link such that more anxious individuals' health outcomes were not buffered when they perceived high social support, whereas less anxious individuals' health did appear to benefit from high support. In other words, social support did not have a salutary influence on the health of more anxious individuals. These effects remained robust when age, relationship length, neuroticism, and marital quality were statistically controlled.

This study is among the first to examine the role of perceived social support in helping to understand the attachment-health link. The finding that health outcomes of more anxious individuals in particular do not seem to be buffered by perceived social support suggests that the mechanism underlying the attachment-health link may be physiological in nature (e.g., impaired immune system functioning). This idea dovetails with existing literature demonstrating the augmented physiological reaction of more anxious individuals to stress [26] and that more anxious individuals have alterations in cellular immunity [6]. Future research, therefore, could fruitfully explore additional possible physiological mechanisms that may explain the relation between attachment anxiety and health outcomes.

Our results also suggest that for some individuals social support may not have salutary influences on their health. Indeed, we found main effects of social support such that some health outcomes (e.g., physical functioning) benefited from greater support, consistent with prior research [9]. We also found an interaction between perceived social support and attachment anxiety such that less anxious individuals experienced better health when they had high support; however, our finding that high or low social support has little influence in the experienced health outcomes of more anxious individuals suggests that the social support-health link may be more nuanced. Our results are consistent with prior research demonstrating that more anxious persons' physiological stress responses (e.g., cortisol reactivity and recovery) are not soothed by higher perceived social support [32], [33]. Indeed, it is important to uncover how and when social support does (and does not) help people experience better health.

This study further extends prior research in its use of health measures; the scales used here investigated more than simple symptom-reporting tendencies or mental health by also examining how current health status may affect physical, role, and social functioning. Our findings thus have implications for understanding the long-term health and functioning of more anxious individuals. If these individuals experience more pain and symptoms indicative of ill health, and feel their health negatively impacts their daily functioning, these effects may in turn impair performance at work or create negative feedback patterns in family life (e.g., more conflict) [23]. To better understand long-term health outcomes, future research should assess health outcomes over time and in response to different life experiences.

Interestingly, no partner effects of attachment or perceived social support emerged in our analyses, consistent with other studies of attachment and perceived social support [33]. An implication of our findings, then, is that self-reported health is primarily associated with individuals' own attachment. It would be premature, however, to cease studying dyads when investigating the attachment-health link. Other research, for instance, suggests that partners can influence each other physiologically. Specifically, Saxbe and Repetti [34] found that if one partner exhibits higher-than-normal cortisol levels at a given time, the other partner is likely to exhibit higher-than-normal cortisol levels as well, suggesting a link between partners' changes in cortisol. Notably, the research by Saxbe and Repetti [34] implemented a longitudinal design, assessed physiological markers of health and stress, and did not take into account partners' attachment orientations. Thus, the lack of partner effects in our study may be sample-specific; it may be that longitudinal studies with physiological health variables provide a better opportunity to observe how romantic partners influence each other's health. It may also be that partner effects on health are attributable to variables other than attachment orientations. These possibilities are readily amenable to future studies.

Before concluding, we note a limitation of the present study. Namely, although the health materials used in the present research are well-validated measures that go beyond the typical self-report of mental health or symptom-reporting, they nonetheless represent self-reports of individuals' health. Our findings are consistent with attachment theory and the current literature on attachment and health, but a more detailed picture will likely emerge from understanding what physically happens in the bodies of more anxious individuals, and then how these physiological processes directly impacts health over time. Future research on attachment and health, therefore, could benefit from using a directly measurable health outcome, such as inflammatory cytokine response or wound-healing [45] or susceptibility to the common cold [46].

In conclusion, a growing body of research is beginning to uncover the biological foundations of attachment processes, such as acute physiological activity in response to stress [25] and how this may influence health for more anxious individuals [12]. The present research represents an important attempt to increase understanding of the relation between individual differences in attachment anxiety and various health outcomes, adding novel insight into the role perceived social support plays in influencing health outcomes for more and less anxious persons. This study thus increases the generalizability of findings on the attachment-health link. Studies on the attachment-health link, nonetheless, are comparatively still in their infancy. Exactly how higher attachment anxiety represents a risk factor for long-term health outcomes is perhaps the next logical step for future research.

## Supporting Information

Table S1 **Results from mixed models statistically controlling for age.**
(PDF)

Table S2 **Results from mixed models statistically controlling for relationship length.**
(PDF)

Table S3 **Results from mixed models statistically controlling for neuroticism.**
(PDF)

Table S4 **Results from mixed models statistically controlling for marital quality.**
(PDF)
