# Supplementary material for: Perceived Social Support Moderates the Link between Attachment Anxiety and Health Outcomes
Source: PLoS One. 2014 Apr 15;9(4):e95358. doi: 10.1371/journal.pone.0095358 (PMC3988242; doi:10.1371/journal.pone.0095358)
Supplement: Table S3 — Results from mixed models statistically controlling for neuroticism. (PDF) [file pone.0095358.s003.pdf]

Supplement to "Perceived Social Support Moderates the Link between Attachment Anxiety and Health Outcomes"

This document contains of the results from our discriminant analyses with neuroticism. The bolded coefficient is the only effect that shifted from significance (p < .05) to a trend (p = .11).

Table S3

Results from Mixed Models with Actor and Partner Scores on Attachment Anxiety and Avoidance, Gender, and Social Support Predicting Health Outcomes, Controlling for Neuroticism

Predictor Variable	Number of Symptoms	Pain	Health Perceptions	Social Functioning	Physical Functioning	Role Functioning	Mental Health
Step 1							
Attachment Anxiety							
Actor	.26**	**.22**	-.21*	-.22*	.03	-.07	.27**
Partner	-.03	-.18	.04	-.05	.07	-.05	.07
Attachment Avoidance							
Actor	-.01	-.11	-.13	.01	-.05	.04	.07
Partner	.05	.13	.04	.10	-.03	.01	-.01
Gender	-.04	-.03	-.03	-.09	-.09**	-.04	-.06
Neuroticism							
Actor	-.36**	-.19	.15*	.08	-.01	.06	-.33**
Partner	-.18*	-.14	.18**	.11	.05	.07	-.07
Step 2							
Social Support							
Actor	-.32	.10	.40	.19	.16	.21	-.39
Partner	-.30	-.13	.21	-.14	.01	.02	-.19

Actor Attachment Anxiety × Actor Social Support	.03	.35	-.56**	-.49*	-.34**	-.35**	-.01
Neuroticism							
Actor	-.36**	-.18	.14*	.07	-.02	.05	-.33**
Partner	-.18*	-.13	.18**	.11	.05	.07	-.07

Note. Reported values are unstandardized regression coefficients. Significance levels are given for each predictor variable at the initial point of entry in the regression equation. Control variables were entered as predictors in both steps.

* $p < .05$, ** $p < .01$
